# From Believing to Doing: The Association Between Leadership Self-Efficacy and the Developmental Leadership Model

**DOI:** 10.3389/fpsyg.2021.669905

**Published:** 2021-08-05

**Authors:** David Bergman, Marie Gustafsson-Sendén, Erik Berntson

**Affiliations:** ^1^Department of Psychology, Stockholm University, Stockholm, Sweden; ^2^Department of Security, Strategy and Leadership, Swedish Defence University, Stockholm, Sweden

**Keywords:** leadership, self-efficacy, developmental leadership, military, training

## Abstract

The current study examined the association between leadership self-efficacy and the developmental leadership model. The purpose was to better understand how leadership training transfers to facets of developmental leadership. This was tested in a cross-sectional design with military commanders in the Swedish Armed Forces. The results show that the sub-domain of leader self-control efficacy (the cognitive and emotional ability to remain composure) did not predict developmental leadership, but that leader assertiveness efficacy (the ability to make rational decisions) predicted the two dimensions of the exemplary model, and inspiration and motivation in the developmental leadership model. One possibility is that leader self-control efficacy can be what enables the individual to function within an extreme context, but leader assertiveness efficacy can be what most determines the leadership performance within that context. The possibility for mediatory analyses in further research is discussed.

## Introduction

Believing that you can do something is not the same thing as actually doing it. Leaders in most organisations will experience a natural gap between training and performance since they will face different social, organisational, and contextual settings when exerting what they have been trained for (Chan and Drasgow, 2001; Porter and McLaughlin, 2006; DeRue and Wellman, 2009; Dragoni et al., 2009). This gap between development and performance can sometimes be bridged by, for example, internships or on-the-job training; but not for uniformed professions like military and law enforcement where the contextual characteristics are hard, not to say impossible, to replicate in training settings (Kolditz, 2007). Thus, a central aspect in such professions is narrowing the training gap as much as possible by increasing the belief of the individual in their own abilities, such as the individual's self-efficacy (Bandura, 1997), as much as possible before facing the demands of the real world.

Individuals having a high belief in their abilities is generally associated with better performance in that specific area or domain (Sadri and Robertson, 1993; Stajkovic and Luthans, 1998; McCormick et al., 2002). One such example is leadership (Hannah et al., 2008), where high beliefs in one's abilities to lead have been found to be associated with more effective leader engagement across varying challenges and the promotion of a more transformational leadership style (Hannah and Luthans, 2008; Yildiz and Simşek, 2016).

Although, *that* a belief in one's ability could be argued to lead to better leader performance, it is not as clear as to exactly *how* this belief will influence the actual performance of leadership. Leadership is a complex interaction between the leader, the subordinates, and the contextual characteristics (Endler and Magnusson, 1976; Hannah et al., 2009), and the research connecting leadership self-efficacy (Hannah et al., 2008) to the specific facets of behaviour in leadership models like transformational leadership (Bass, 1999) or developmental leadership (Larsson et al., 2003) are limited.

### Leadership Self-Efficacy

Leadership self-efficacy is a specific form of efficacy associated with the level of confidence in the knowledge, skills, and abilities associated with leading others (Hannah et al., 2008). It is a domain-specific construct that refers to the “beliefs in one's capabilities to organise and execute the courses of action required to produce given attainments” a person has (Bandura, 1997, p. 3). Self-efficacy can transfer across highly disparate domains under certain mechanisms, referred to as “domain transfer.” Bandura (1997) demonstrated that strong personal triumphs are likely to affect this transformational restructuring of self-efficacy beliefs as the result of a “powerful mastery experience” (p. 53). Transfer of self-efficacy is, therefore, a central mechanism in preparing future military officers to lead in combat. The strong personal triumph in mastering one difficult task (demanding training course) can create an increased individual belief that other tasks with equal or greater difficulty (leading in combat) can be overcome in the same way (Samuels and Gibb, 2002; Samuels et al., 2010).

We argue that military training courses that present a perceived threat to life and require active mastery have shown to affect leadership self-efficacy within the two subcomponents of leader self-control efficacy (to maintain cognitive and emotional control) and leader assertiveness efficacy (the ability to make immediate and correct decisions in leading others) (Samuels et al., 2010; Bergman et al., 2019). The cognitive and emotional ability to maintain composure (self-control efficacy) and the ability to make rational decisions (leader assertiveness efficacy) are key dimensions for leaders in extreme contexts (Kolditz, 2007). These sub-dimensions also correspond with other conceptualizations of leadership self-efficacy (Hannah et al., 2008). Leader self-control efficacy can contribute to efficacy for thought, self-motivation, and action, whereas leader assertiveness efficacy can contribute to efficacy for means and action (Samuels et al., 2010).

### Developmental Leadership

Leadership refers to the ability of an individual to influence, motivate, and enable others to contribute towards the effectiveness and success of the organisations of which they are members (House et al., 1999; Bass, 2008). In this definition, leadership not only focuses on the actions of the individual leader, but also the interaction between a number of contextual and organisational characteristics.

The leadership model used by the Swedish Armed Forces is the developmental leadership model (DL) (Larsson et al., 2003; Larsson, 2006). This model builds upon transformational leadership (TL) (Bass, 1999) including the full range leadership model (Avolio, 1999; Bass, 1999), where leaders act in such a way that they enhance the motivation, morale, and job performance of their followers.

Indeed, both TL and DL present a hierarchy of leadership behaviours (Bass and Bass, 2008; Larsson et al., 2018). *The first*, and least desired form of leadership, is *laissez-faire*, or non-leadership. The *laissez-faire* is defined as the absence of leadership, where leaders avoid making decisions and reflect avoidance and withdrawal from leadership duties. *The second* is the conventional (or transactional) leadership style. Conventional leadership relies on contingent reward where the leader exercises a high degree of control in relation to their subordinates. It is primarily concerned with formal agreements where the leader hands out tasks and the subordinates execute them because that is what the hierarchy and organisation stipulate, often motivated primarily by reward or threats of sanctions or punishment. *The third* is the developmental leadership style where the behaviour of the leader inspires subordinates to perform beyond their perceived abilities in a way that improves both the individuals and the organisation, hence the transformation or development implied by the name. In the developmental leadership model, it is also argued that contextual and individual characteristics influence the leadership style. Contextual characteristics are reflected through the external environment, organisational structure and culture, and dynamics of the group, whereas individual characteristics are divided into basic prerequisites (physical ability, physiological, view of life) and desirable competencies (task-related competence, management-related competence, social competence, and the ability to cope with stress). Each of the four competencies has shown to be related to a more developmental leadership style (Larsson et al., 2003; Larsson, 2006).

The differences between TL and DL are mostly oriented around cultural variations, where DL has been adjusted with research within a Scandinavian context. For example, the element of charisma, central to transformational leadership (Bass, 1999), was found unsuitable in a Scandinavian leadership culture (Larsson et al., 2003).

The developmental leadership style could be effective in military settings for several reasons. A developmental approach can motivate, stimulate, and inspire subordinates to perform beyond their own perceived abilities in a context where their wellbeing might be dependent on it (Larsson et al., 2018). When serving in life-threatening contexts, the transactional incentives of conventional leadership (pay, reward, or punishment) are inadequate (Kolditz, 2007). Pay and rewards lose their motivational value if the subordinate might not live to enjoy them, and threats of administrative punishment have little incentive when the alternative is injury or death. Subordinates in such circumstances must be led in ways that inspire trust and confidence, which then develops (or transforms) followers into willing, rather than compliant agents (Kolditz, 2007). The limitation with a mere conventional style of leadership is that it often relies too heavily on control and, at best, reaches the objectives that are demanded. Transactional leadership can be sufficient and positive in a context with a given framework and clear requirements on the performance of subordinates. But for the same reason, it is often inadequate in an extreme setting where the contextual frame is changeable and a greater degree of autonomy is required of subordinates (Lim and Ployhart, 2004; Sweeney, 2010). This is not to say that traditional incentives like pay, promotions, and medals are unimportant in an extreme setting—just less important.

### The Association Between Leadership Self-Efficacy and Developmental Leadership

Leadership self-efficacy could be associated with the facets of developmental leadership in several ways. From a general perspective, self-efficacy can facilitate cognitive control and functioning in stressful situations. Furthermore, leadership self-efficacy may reduce the effects of stress and allow the leader to focus on leading subordinates towards a common goal rather than worrying about the potential drawbacks of a difficult situation (Murphy and Ensher, 1999). Self-efficacy has also been associated with the ability of the individual to maintain cognitive abilities despite difficulties, obstacles, and disappointments (McCormick et al., 2002; McCormick and Martinko, 2004). Likewise, in DL, the ability to manage stress is associated with effective developmental leadership by mastering one's own regulations under stress (intrinsic regulation) while being able to act in a way that enables subordinates to manage their feelings (extrinsic regulation) (Larsson et al., 2018).

Self-efficacy can also influence the individual selection of leadership strategy. Leadership self-efficacy is critical to the leadership process because it affects the development and execution of the strategies and goals of the leader for any given leadership situation (McCormick, 2001). This is consistent with Wood and Bandura (1989) findings that self-efficacy will shape the selection of strategy for a specific situation (like leadership) by an individual. Thus, higher leadership self-efficacy has been connected to higher levels of transformational leadership (Chemers et al., 2000; Luthans and Peterson, 2002; Dvir and Shamir, 2003; Finn et al., 2007), and vice versa; as leaders with low leadership self-efficacy can be more likely to adopt a *laissez-faire* leadership style (Courtright et al., 2014). In other words, individuals that are more confident in their ability to lead will also be more transformational/developmental in their leadership style.

Leadership self-efficacy could also influence the three dimensions that constitute developmental leadership directly. At the core of each leadership style, certain facets of leadership behaviours can be found, and when these are combined, a leadership style is created (Larsson et al., 2018). Thus, leadership self-efficacy may influence specific dimensions that comprise a developmental leader.

*Exemplary model* is the first dimension and relies more on a state of mind rather than behaviours, being characterised by self-reliance and a morally good, “live as you learn”-mindset (Larsson et al., 2018). Kolditz (2007) summarised the need for leaders to act as role models, in that: “a leader who appears confident sends a tacit message to subordinates: that they should rely on the leader's competence because the leader is convinced it exists” (p. 75). Liden and Mitchell (1988) suggest that efficacious leaders engage in ingratiatory behaviours in order to present a favourable image to subordinates and act in a way that conveys confidence. A leader high in self-efficacy (i.e., more reliant on their abilities) will likely present more authentic behaviour and exhibit a high standard of ethics. In effect, their approach to subordinates can be more genuine. Additionally, they can act credibly based on authenticity and act as role-models according to moral codes. This involves having the integrity to stand by convictions even when they may not be popular, and assert these when put in leadership situations. Authentic elements of leadership are vital to the influence of the leaders on ethical values promoted towards the subordinates and the organisation (Hattke and Hattke, 2019).

*Individualised consideration* is the second dimension of DL, and means providing support and interactions that make subordinates feel important and competent, which, in turn, increases their potential for performance and development (Larsson et al., 2018). When leaders exhibit genuine concern for subordinates, the followers tend to raise their own self-efficacy, and in turn, appear more eager and perform assigned tasks with a higher level of commitment. In effect, this increases the liking the leader has for subordinates and perceived similarity, which then reinforces their trust in subordinates and efficacious beliefs in their own ability to lead, and the cycle begins again (Wayne and Liden, 1995). Granted, such reasoning is dependent on the long-term relationship between the leader and the subordinate, and little is known about how the mechanism of leadership self-efficacy will affect this dimension at an early stage.

*Inspiration and motivation* is the third and last dimension. It refers to the different ways a leader acts inspiring for their subordinates, which promotes both participation and a common understanding of higher objectives (Larsson et al., 2018). Leadership self-efficacy has been shown to affect communication and encouragement between leaders and followers in a positive way (Mellor et al., 2006). It may also predict the persistence of a leader in trying to persuade others when in a leadership position (Savard and Rogers, 1992). This is consistent with the theory of emotional and behavioural contagion in leadership, where the emotional state of a leader influences how their subordinates feel (Johnson, 2008). The approach of the leader may both produce and reinforce positive and negative feelings within subordinates. For example, a leader who has doubts about his abilities may diminish or pacify the most enthusiastic employee, while a self-efficacious leader may inspire involvement.

Regarding the sub-dimensions of leadership self-efficacy, there is insufficient research to hypothesise regarding whether these sub-dimensions have an equal or disproportionate effect on the behavioural facets of leadership. Leader self-control efficacy can contribute to efficacy for thought, self-motivation, and action, whereas leader assertiveness efficacy can contribute to efficacy for means and action (Samuels et al., 2010). As such, both sub-dimensions can arguably affect the dimensions of the developmental leadership model presented above.

### The Present Study

The aim of the present study was to investigate how leadership self-efficacy, in terms of leader self-control efficacy and leader assertiveness efficacy, is associated with the three dimensions in the developmental leadership model. Based on the reasoning above, we hypothesised that leadership self-efficacy (indicated by leader self-control efficacy and leader assertiveness efficacy) is positively associated with all subdimensions of developmental leadership (indicated by exemplary model, individualised consideration, and inspiration/motivation).


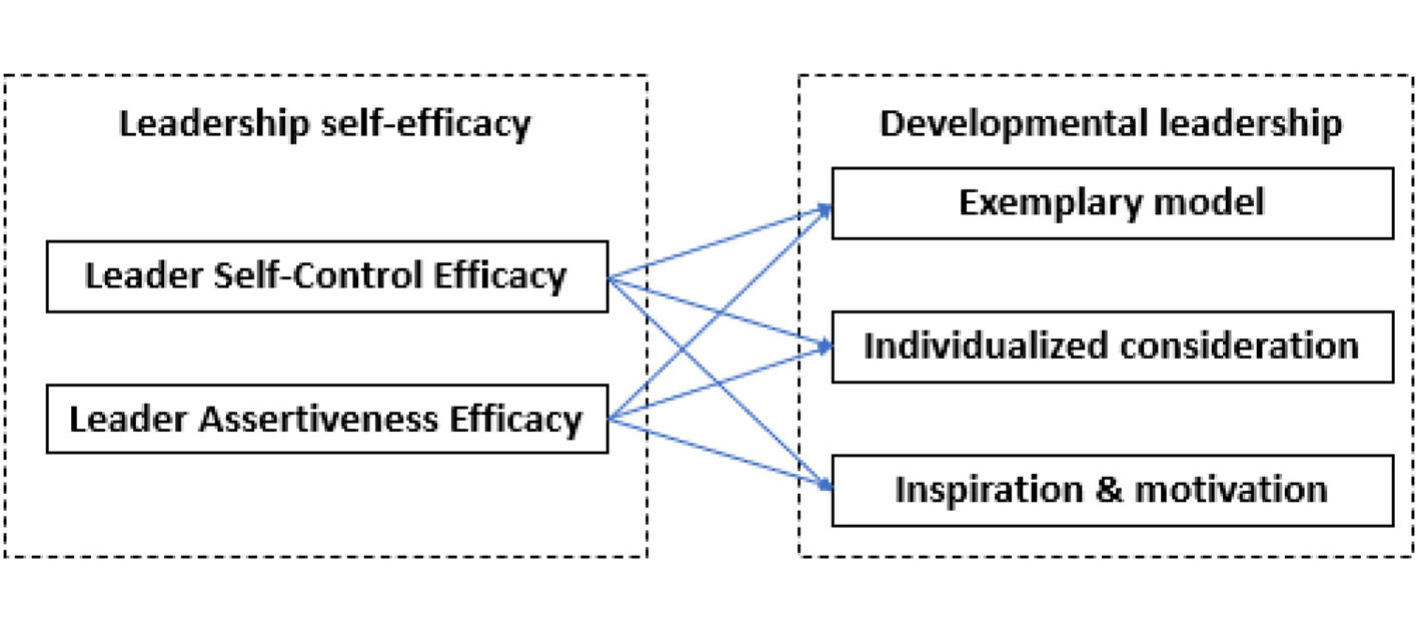


## Method

The study with its procedures and measures received ethical approval from the Swedish Ethical Review Authority, reference number 2019-03118. Participants were informed of the parameters, procedure, and voluntariness of the study and gave their informed consent. No compensation was offered for participation.

### Participants

The participants were 111 military students and teachers from the Swedish Defence University (women = 11%, men = 89%, M_age_ = 47.77 years at the time each respondent started the course, SD_age_ = 8.65). They were enrolled students or teachers in the Higher Officer Training Programme required for promotion to the rank of OF3 (major/lieutenant commander) and OF4 (lieutenant colonel/commander), and came from all branches of the armed forces (Army 67%, Navy 21%, Air Force 9%, other 3%). Individuals qualify by prior service at mid-level leadership positions like company commanders/deputy in the Army, squadron commanders/deputy in the Air Force, or ship's captain/executive officer in the Navy. They applied to the programme voluntarily, and each unit ranked applicants based on previous performance and prognosis for higher command positions. They had a mean of 24.6 years of prior service, *SD*_*service*_ = 9.7. The majority (74.6%) had conducted at least one operational deployment defined as permanent service in an area of operations lasting at least 2 months or longer (*M*_*deployment*_ = 1.75, *SD*_*deployment*_ = 1.84, range from 0 to 10). Of those who had conducted at least one deployment or more, 21.6% had been in a combat situation defined as a confrontation with opposing forces with a threat to life. Of all participants, 45.9% had completed the basic airborne course and were parachute qualified.

### Procedure

Participants were contacted with the internal email send list for the Higher Officer Training Programme and the list of addresses issued to each student and teacher assigned to the Swedish Defence University. The email contained information about the study and a link to the survey. A total of 225 potential participants were contacted. It is unknown how many of these were absent for natural reasons (voluntary withdrawal, exchange programs abroad, etc.). Out of the 225 individuals contacted, 117 responded. Of the 117 responses, six were incomplete, which left us with 111 participants included in the analysis. Questions were self-assessments of the most recent leadership position of the individuals before commencing training.

### Measures

*Leadership self-efficacy* was measured using a short version of the leadership self-efficacy scale (LSES) with the subscales of leader self-control efficacy and leader assertiveness efficacy (Samuels et al., 2010; Bergman et al., 2019). The subscales had items such as “I can easily shift attention away from thoughts that scare me” (self-control) and “I can easily lead others, maintain the same high standards, and not be seen as hypocritical” (assertiveness). Respondents marked their answers on a seven-point response scale (1 = do not agree, 7 = do fully agree). Cronbach's alpha in the present study was: 0.81 for leader self-control efficacy and 0.71 for leader assertiveness efficacy.

*Developmental leadership* was measured using the developmental leadership questionnaire (DLQ) with the three dimensions of exemplary model, individualised consideration, and inspiration and motivation (Larsson et al., 2003; Larsson, 2006). The dimensions had items such as “Discusses what values are important before making decisions” (exemplary model), “Show empathy for people's needs” (individualised consideration), and “create enthusiasm for a task” (inspiration and motivation). Respondents marked their answers on a on a nine-point response scale (1 = do not agree, 9 = do fully agree). Cronbach's alpha in this study was: 0.77 for exemplary model, 0.84 for individualised consideration, and 0.80 for inspiration and motivation.

Four desirable competencies for developmental leadership were also included in the questionnaire (*task-related competence, management-related competence, social competence*, and *the capacity to cope with stress*). The competencies had items such as “Shows proficiency within the unit's occupational area” (task-related competence), “Makes sure the subordinates are informed” (management-related competence), “Easily connects with others” (social competence), and “Acts calm towards others in situations of stress” (capacity to cope with stress). Respondents marked their answers on a nine-point response scale (1 = do not agree, 9 = do fully agree). Cronbach's alpha in this study was: 0.76 for task-related competence, 0.75 for management-related competence, 0.78 for social competence, and 0.88 for capacity to cope with stress.

## Analysis

Initially, assumptions of normality and homoscedasticity were tested by means of analysing a probability plot of residuals (P–P plot), and a scatterplot was produced. A visual examination of both plots showed that the criteria for the assumptions of normality and homoscedasticity were met. To determine the risk of multicollinearity and establish that each of the variables would provide unique or independent information in the subsequent regression models, the variance inflation factor (VIF) was calculated for all variables. The results were in the range 1.1–2.2, which falls within the threshold range of 0.2–4 (Hair et al., 2010), indicating that multicollinearity would not be a problem in the regression models.

As a second step, the dimensionality of the study scales (*leader self-control efficacy, leader assertiveness efficacy, exemplary model, individual consideration, inspiration and motivation, task-related competence, management-related competence, social competence, and capacity to cope with stress*) was tested by means of a Confirmatory Factor Analysis (CFA). Three models—namely, a null model, a one-factor model, and our hypothesised 9-factor model—were tested by loading items on nine separate factors. Model selection was based on fit indices Chi square test (χ2), Root Mean Square Error of Approximation (RMSEA), Standardised Root Mean Square Residual (SRMR), Comparative Fit Index (CFI) and Tucker Lewis index (TLI). Results from the CFA are reported in Supplementary Tables 1, 2, which displays factor loadings and model fit. The results indicate an acceptable fit to data, although the loadings and model fit also indicate some instability in the measures at hand.

In order to test the association between the two dimensions of self-efficacy and the three dimensions of developmental leadership, we calculated three separate hierarchical multiple regressions for each of the three dimensions (exemplary model, individualised consideration, and inspiration and motivation) of developmental leadership. The regressions were computed in three steps to clarify the addition of variance in each step. Step one included the basic variables of age and gender and if the individual had completed basic parachute training, step two added the two subcomponents of leadership self-efficacy, and step three added the four desirable competencies from the developmental leadership model.

## Results

Correlations, means, and standard deviations are presented in Table 1. The regressions with the contribution of variance in each step can be seen in Table 2.

**Table 1:** Correlations, means, and standard deviations.

	**1**	**2**	**3**	**4**	**5**	**6**	**7**	**8**	**9**	**10**	**11**			**N**	**M**	**SD**
1	Leader self-control efficacy	-												111	5.24	0.93
2	Leader assertiveness efficacy	0.466^***^	-											111	5.66	0.79
3	Exemplary model	0.356^***^	0.451^***^	-										111	7.84	0.59
4	Individual consideration	0.218^*^	0.245^**^	0.656^***^	-									111	7.21	0.90
5	Inspiration and motivation	0.259^**^	0.435^***^	0.669^***^	0.576^***^	-								111	7.41	0.76
6	Task related competence	0.092	0.292^***^	0.331^***^	0.177	0.464^***^	-							111	7.67	1.13
7	Management related competence	0.172	0.221^*^	0.506^***^	0.351^***^	0.592^***^	0.559^***^	-						111	7.39	0.97
8	Social competence	0.087	0.309^**^	0.369^***^	0.551^***^	0.559^***^	0.379^***^	0.464^***^						111	7.67	1.14
9	Capacity to cope with stress	0.335^***^	0.499^***^	0.497^***^	0.335^***^	0.639^***^	0.474^***^	0.569^***^	0.460^***^					111	7.33	1.03
10	Age	0.081	0.151	0.340^***^	0.185	0.118	0.104	0.182	−0.086	0.046	-			111	47.76	8.64
11	Gender	−0.023	−0.039	0.075	−0.078	0.101	0.272^**^	0.341^***^	−0.064	0.141	0.135	-		111	1.89	0.31
12	Parachute training	0.160	0.92	0.174	0.240^*^	0.139	0.034	−0.049	0.201	0.064	−0.025	−0.90		−89	1.43	0.497

* *p < 0.05*,

** *p < 0.01*,

*** *p < 0.001*.

**Table 2:** The regressions with the contribution of variance in each step.

	**Exemplary model**			**Individual consideration**			**Inspiration and motivation**		
	**Step 1**	**Step 2**	**Step 3**	**Step 1**	**Step 2**	**Step 3**	**Step 1**	**Step 2**	**Step 3**
	**β**	**β**	**β**	**β**	**β**	**β**	**β**	**β**	**β**
Age	0.404^***^	0.326^***^	0.309^***^	0.236	0.188	0.247^**^	0.085	0.006	0.025
Gender	−0.016	0.029	−0.037	−0.080	−0.052	−0.062	0.095	0.139	0.006
Parachute-training	0.182	0.122	0.130	0.239	0.205	0.136	0.149	0.103	0.082
Leader self-control efficacy		0.117	0.110		0.047	0.114		−0.002	0.005
Leader assertiveness efficacy		0.473^***^	0.378^***^		0.303^**^	0.092		0.530^***^	0.186
Task related competence			0.004			−0.041			0.099
Management related competence			0.211			0.099			0.256^*^
Social competence			0.119			0.432^***^			0.170
Capacity to cope with stress			0.025			−0.044			0.230^*^
Δ*R*^2^	0.191^***^	0.281^***^	0.042^***^	0.113	0.104^*^	0.159^***^	0.038	0.270^***^	0.227^***^
Total *R*^2^			0.514^***^			0.321^***^			0.535^***^

* *p < 0.05*,

** *p < 0.01*,

*** *p < 0.001*.

*β, Regression coefficient, R^2^, Coefficient of determination*.

For *exemplary model*, the basic variables explained 19.1% of the variance in the first step. In this step, age was associated with the outcome, where older respondents reported higher levels of exemplary model. In the second step, leadership self-efficacy added 28.1% of the variance. Specifically, higher leader assertiveness efficacy was significantly associated with exemplary model, and leader self-control was also positively associated but at a lower level of significance. The third and last step added 0.42% explained variance. Leader assertiveness efficacy and age remained significant predictors.

For *individualised consideration*, the basic variables explained 11.3% of the variance in the first step. No variable was significant at this step. The second step with leadership self-efficacy added 10.4% of the variance. Specifically, leader assertiveness efficacy was a significant predictor. The third and last step added 15.9%, and social competency and age were significantly associated to the dimension. At this third step, leader assertiveness efficacy was no longer significant.

For *inspiration and motivation*, the basic variables explained 3.8% of the variance and no variable was significantly associated with the outcome. The second step with leadership self-efficacy explained 27.0% of the variance. Specifically, higher leader assertiveness efficacy was significantly associated with this specific leadership dimension, whereas self-control was not. The third and last step added 22.7% explanation for the variance. Management-related competence and the capacity to cope with stress were significant predictors. At this third step, leader assertiveness efficacy was no longer significant.

## Discussion

The aim of the present study was to investigate how leadership self-efficacy (indicated by leader self-control efficacy and leader assertiveness efficacy) was associated with developmental leadership and its specific dimensions. The results indicated partial associations between the study variables.

Leader self-control efficacy did not predict developmental leadership in any dimensions. Although it had a strong correlation with the other subdimension of leadership self-efficacy, it did not contribute with any unique variance in any of the dimensions of developmental leadership.

Leader assertiveness efficacy predicted three dimensions of developmental leadership. When the desirable competencies from the developmental leadership model were added, the beta-weights for leader assertiveness efficacy ceased being significant or remaining significant but with a lower beta coefficient in the two dimensions of individual consideration and inspiration and motivation. This is not that surprising given that the four factors added are those that have previously been shown to contribute to developmental leadership (Larsson, 2006), indicating that leadership assertiveness efficacy alone is not sufficient but requires specific competencies for successful leadership performance.

The differences between leader self-control efficacy and leader assertiveness efficacy are particularly interesting. Leader self-control efficacy did not seem to contribute directly to the three dimensions of developmental leadership as has been suggested. One possible explanation is that leader self-control efficacy is more of a prerequisite than something that directly affects leadership behaviours. This explanation is supported by self-efficacy theory, in that the individual ability to maintain cognitive, emotional, and behavioural regulation *allows* assertive action, leading to successful performance (Bandura, 1997). The definitions of the sub-components by Samuels et al. (2010) also imply that leader assertiveness efficacy is more closely related to action and performance when they describe that “self-control efficacy can contribute to efficacy for thought, self-motivation, and action, whereas leader assertiveness efficacy can contribute to efficacy for means and action” (p. 121). Simplified, leader self-control efficacy can be what enables the individual to function within an extreme context, whereas leader assertiveness efficacy can be what most determines the leadership behaviour within that context.

Another possibility is that leadership self-efficacy and the four desirable competencies are not equal predictors to the facets of developmental leadership, and that a mediation analysis might offer further insight. Although the present sample is cross-sectional and also too small for structural equation modelling, such methods could help build a conceptual model with a hypothesised mediation process to better understand the associations between leadership self-efficacy, desirable competencies, and the facets of developmental leadership.

One interesting finding was that age was a significant predictor in two of the three dimensions, while having undergone parachute training did not predict any dimensions of developmental leadership. Although previous research has shown that such training can transfer to leadership self-efficacy (Bergman et al., 2019), it does not seem to be as directly related to specific leadership behaviours as previously assumed, whereas age indicating experience as a leader could be assumed to be more relevant. In this respect, it has been found that experience and previous performance are important primary sources of self-efficacy (Sitzmann and Yeo, 2013). Experience can also be a factor that increases motivation and limits “reality shock” when individuals confront the extreme stress of warfare for the first time (Brænder and Andersen, 2013). Thus, one possible interpretation of the results in the present study is that specific training courses can indeed increase self-efficacy in the short term; but when utilised in performance and experience, these become the primary predictors of developmental leadership. Although the argument for experience includes a catch 22: one does not get experienced without experiencing something for the first time. A practical implication from this could be that specific training courses are indeed necessary, but future leaders also need assistance in processing the short-term effects of training more directed towards specific leadership situations before encountering them in life-threatening situations. Another interesting finding in the present study concerns the background variable of gender, which did not predict any facets of developmental leadership. Gender was not included in any hypothesis, however the total absence of significant results is surprising, and the results (or absence thereof) are contradictory to what one might expect. Previous research has studied gender differences in leadership self-efficacy, specifically Eibl et al. (2020) and Robinson et al. (2016), as well as in related fields, such as academic self-efficacy and performance in school settings (Huang, 2013) and sport self-efficacy and athletic performance (Lirgg, 1992). One possible explanation for the absence of differences in this specific field is that the socialisation process could have been stronger in the development of military leaders to reduce the impact of individual characteristics (Dalenberg and Buijs, 2013). Another possibility is that those who select to enter and remain in military service do so to a greater extent based on certain values and beliefs (Bachman et al., 1987), possibly contributing to a more homogenous sample by self-selection.

### Limitations

Apart from the limited possibilities of a mediation analysis, the present study presents four primary limitations worth mentioning. First, the study used a cross-sectional design that limited casual attributions and offered some limitations in interpreting the results. Longitudinal methods of assessment could offer further insight. Second, the present study rated leadership from self-assessments of performance, and not the more comprehensive 360°-rating (assessment of leadership made both by individual as well as subordinates, peers, and superior commanders) presented by Larsson (2006). Previous research has shown that leaders tend to over-estimate specific behaviours such as transformational leadership (Lee and Carpenter, 2018). Using the full range of the DLQ could offer insight in further studies, as well as using multimode sources of feedback, focusing on the leader's self-awareness, and “other-awareness” of their subordinates (Vogel and Kroll, 2019). Third, the sampling point did not occur at a time when the respondents were in a leadership position, introducing the risk of a recall bias. Sampling of leaders in an actual command position could have offered further insight. However, it is worth pointing out that all participants—both student and teachers—have qualified themselves for the positions through previous command in the military organisation, and were selected for higher training based partly on leadership skills, making them relevant in the sample. Fourth, the confirmatory factor analysis indicated a somewhat unstable factor structure, and it may mean that other competing models would show better fit to the data. Although, a few significant items scored quite low, and the RMSEA was acceptable (just under 0.10) even if it did not showing a very good fit to data, which may have affected the results of the analyses in the study. We chose to retain the mean values indexes instead of using the factor scores to impute our composite variables in order to have more comparable measures in relation to other studies using the same model (Larsson, 2006). Despite this limitation, all factors discriminated to each other and the hypothesised structure was reproduced in data, which is why we also believe that the measures were acceptable.

## Data Availability Statement

The raw data supporting the conclusions of this article will be made available by the authors, without undue reservation.

## Ethics Statement

The studies involving human participants were reviewed and approved by Swedish ethical review authority, reference number 2019-03118. The patients/participants provided their written informed consent to participate in this study.

## Author Contributions

All authors listed have made a substantial, direct and intellectual contribution to the work, and approved it for publication.

## Conflict of Interest

The authors declare that the research was conducted in the absence of any commercial or financial relationships that could be construed as a potential conflict of interest.

## Publisher's Note

All claims expressed in this article are solely those of the authors and do not necessarily represent those of their affiliated organizations, or those of the publisher, the editors and the reviewers. Any product that may be evaluated in this article, or claim that may be made by its manufacturer, is not guaranteed or endorsed by the publisher.
