# Supplementary material for: From Believing to Doing: The Association Between Leadership Self-Efficacy and the Developmental Leadership Model
Source: Front Psychol. 2021 Aug 5;12:669905. doi: 10.3389/fpsyg.2021.669905 (PMC8374048; doi:10.3389/fpsyg.2021.669905)
Supplement: Supplementary file 1 [file Table_1.DOCX]

Table A1. Fit statistics for tested models.

| Model | df | $\chi^{2}$ | p | RMSEA | SRMR | CFI | TLI | Model | $\Delta$df | $\Delta\chi^{2}$ | p |
| --- | --- | --- | --- | --- | --- | --- | --- | --- | --- | --- | --- |
| 0. Null model | 703 | 3022.7 | .000 |  |  |  |  |  | - | - | - |
| 1. 1 factor | 665 | 1839.0 | .000 | .127 | .11 | .49 | .47 | 1 vs 0 | 38 | 1183.7 | .000 |
| 2. 9 factors | 629 | 1310.4 | .000 | .099 | .11 | .71 | .67 | 2 vs 1 | 36 | 528.6 | .000 |

Notes: N *=* 110, out of 111 in the sample.

Table A2. Study scales, items and factor loadings from Confirmatory factor analysis

|  |  | Item | Factor |
| --- | --- | --- | --- |
|  | LSE Self-Control | *LSE Self-Control* |  |
| LSES03 |  | I can easily shift attention away from thoughts that scare me | .77 |
| LSES09 |  | I can easily distract myself from thoughts that might interfere with my task at hand | .77 |
| LSES12 |  | I am always able to perform despite any fear brought on by thoughts that are frightening | .76 |
|  |  |  |  |
|  | LSE Assertiveness | *LSE Assertiveness* |  |
| LSES08 |  | I can set a personal example for colleagues and subordinates | .59 |
| LSES10 |  | I can easily confront my peers when they go against regulation and correct or report them if need be | .69 |
| LSES11 |  | I can easily lead others, maintain the same high standards, and not be seen as hypocritical | .77 |
|  |  |  |  |
|  |  |  |  |
|  | Föredöme | *Exemplary Model* |  |
| X1 | Jag diskuterar inför beslut vilka värden som är viktiga | I discuss what values are important before making decisions | .60 |
| X2 | Jag uppvisar ett etiskt och moraliskt förhållningssätt | I display an ethical and moral attitude | .49 |
| X3 | Jag ger uttryck för värderingar som vilar på humanistisk grund | I express values that have a humanistic basis | .22 |
| X4 | Jag handlar i överensstämmelse med mina uttryckta åsikter | I act in accordance with the opinions I express | .59 |
| X5 | Jag engagerar medarbetarna i verksamhetens övergripande målbilder och visioner | I engage my co-workers in the organization’s goals and visions. | .65 |
| X6 | Jag erkänner egna misstag utan att komma med bortförklaringar | I admit my own mistakes without trying to make excuses | .56 |
| X7 | Jag tar ansvar för verksamheten – även i motgång | I accept responsibility for the operations – even in hard times | .63 |
| X8 | Jag tar ansvar för medarbetarnas välmående | I accept responsibility of my co-workers wellbeing. | .53 |
| X9 | Jag tar ansvar för att påbörjade uppgifter fullföljs | I accept responsibility to ensure  that started tasks are completed | .55 |
|  |  |  |  |
|  | Personlig | *Individualized Consideration* |  |
| X10 | Jag visar inlevelse i människors behov | I show insight into people’s need | .69 |
| X11 | Jag tar mig tid att lyssna | I take time to listen | .66 |
| X14 | Jag beaktar medarbetarnas synpunkter | I consider co-workers opinions | .48 |
| X15 | Jag handskas bra mer personer som ej utfört sin uppgift väl | I treat people appropriately who have not carried out tasks well | .75 |
| X16 | Jag tar itu med relationsproblem | I tackle relationship problem | .76 |
| X17 | Jag kan hantera besvärliga medarbetare | I can deal with troublesome co-workers | .76 |
|  |  |  |  |
|  | Inspiration | *Inspiration and motivation* |  |
| X18 | Jag skapar entusiasm inför en uppgift | I create enthusiasm for a task | .70 |
| X20 | Jag bidrar till en arbetsglädje som får andra att anstränga sig mer | I contribute to a work climate (job satisfaction) that encourages others to work harder | .74 |
| X21 | Jag får andra att känna ansvar för enhetens framtida mål | I can make others to share  responsibility for the unit’s  future goals | .71 |
| X23 | Jag inspirerar andra till kreativt tänkandeI | I inspire others to think creatively | .80 |
| X24 | Jag inspirerar andra till att pröva nya sätt att arbeta | I inspire others to try new working  methods | .75 |
| X25 | Jag strävar efter att uppnå överenskommelser om vad som skall göras | I aim to reach agreements on what shall be done | .23 |
|  |  |  |  |
|  | Fackkompetens | *Task-related competence* |  |
| X55 | Jag följer med intresse utvecklingen inom enhetens yrkesområden | I follow the development within my area of work with interest | .80 |
| X56 | Jag visar prov på kunnighet inom enhetens yrkesområde | I demonstrate competence within my area of work | .80 |
|  |  |  |  |
|  | Chefskompetens | *Management-related competence* |  |
| X57 | Jag följer upp hur verksamhetens mål nås | I follow up how the operation’s goals are attained | .37 |
| X58 | Jag ser till att medarbetarna hålls informerade | I ensure that co-workers are kept informed | .36 |
| X59 | Jag framför min syn på vad skeenden i omvärlden betyder för den egna organisationen | I express my opinion on what external events mean for our own organization | .92 |
| X60 | Jag upptäcker tidigt förhållanden i omvärlden som kan påverka den egna verksamheten | I am quick to discover external conditions that may affect our organization | .90 |
|  |  |  |  |
|  | Social kompetens | *Social competence* |  |
| X61 | Jag får lätt kontakt med andra | I communicate easily with others | .92 |
| X62 | Jag är flexibel i kontakt med andra – ”pratar med bönder på bönders vis och med lärde på latin” | I am flexible in contact with others adapt my communication to different people | .70 |
|  |  |  |  |
|  | Stresstålighet | *Capacity to cope with stress* |  |
| X63 | Jag agerar lugnt mot andra i stressfyllda situationer | I act calm in stressful situations. | .83 |
| X65 | Jag fattar bra beslut under press, även om informationen är ofullständig | I make good decisions under pressure, even when lacking full information | .86 |
| X66 | Jag är bra på att handskas med diffusa och oklara lägen | I am good at handling with diffuse and unclear situations | .84 |

All factor loadings significant in CFA, where p>.001 for loadings >.XX and p>.05 for loadings >.XX.

N = 110, out of 111 in the sample.
